# Botanical Sources, Chemistry, Analysis, and Biological Activity of Furanocoumarins of Pharmaceutical Interest

**DOI:** 10.3390/molecules24112163

**Published:** 2019-06-08

**Authors:** Renato Bruni, Davide Barreca, Michele Protti, Virginia Brighenti, Laura Righetti, Lisa Anceschi, Laura Mercolini, Stefania Benvenuti, Giuseppe Gattuso, Federica Pellati

**Affiliations:** 1 Department of Food and Drug, University of Parma, Parco Area delle Scienze 27/A, 43124 Parma, Italy; renato.bruni@unipr.it (R.B.); laura.righetti@unipr.it (L.R.); 2 Department of Chemical, Biological, Pharmaceutical and Environmental Sciences, University of Messina, Viale F. Stagno d’Alcontres 31, 98166 Messina, Italy; dbarreca@unime.it (D.B.); ggattuso@unime.it (G.G.); 3 Department of Pharmacy and Biotechnology (FaBiT), Alma Mater Studiorum, University of Bologna, Via Belmeloro 6, 40126 Bologna, Italy; michele.protti2@unibo.it (M.P.); laura.mercolini@unibo.it (L.M.); 4 Department of Life Sciences, University of Modena and Reggio Emilia, Via G. Campi 103, 41125 Modena, Italy; virginia.brighenti@unimore.it (V.B.); lisa.anceschi@gmail.com (L.A.); stefania.benvenuti@unimore.it (S.B.)

**Keywords:** furanocoumarins, plants, botany, chemistry, extraction, analysis, biological activity

## Abstract

The aim of this work is to provide a critical review of plant furanocoumarins from different points of view, including their chemistry and biosynthetic pathways to their extraction, analysis, and synthesis, to the main biological activities found for these active compounds, in order to highlight their potential within pharmaceutical science. The limits and the possible improvements needed for research involving these molecules are also highlighted and discussed.

## 1. Biosynthesis of Furanocoumarins

**Figure 1:**
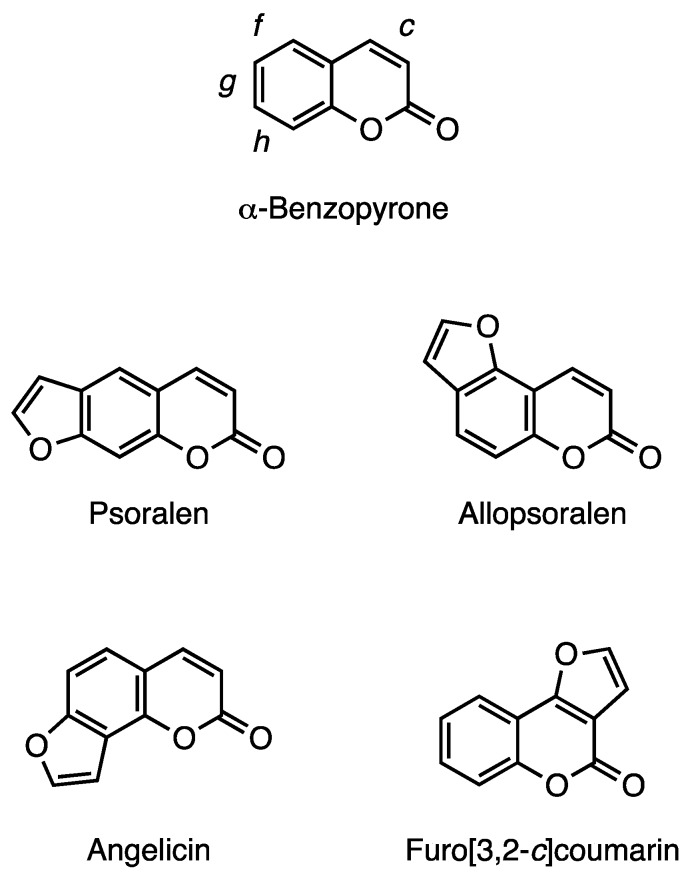
Core structure of the most common naturally occurring furocoumarin derivatives.

Furanocoumarins are tricyclic aromatic compounds composed of a furan ring fused to a α‑benzopyrone (coumarin) system. Even though the furan moiety can be in either a 2,3- or 3,2-arrangement at the *c*, *f*, *g*, or *h* bonds of the coumarin, most naturally occurring derivatives belong to the psoralen (furo [3,2-*g*][1]benzopyran-7-one), allopsoralen (furo[2,3-*f*][1]benzopyran-7-one), angelicin (furo[2,3-*h*][1]benzopyran-7-one), and furo[3,2-*c*]coumarin (furo[3,2-*c*][1]benzopyran-7-one) subclasses (Figure 1). The whole class can be structurally divided into angular and linear furanocoumarins, with the latter being compounds with the furan ring attached at the 6,7 positions on the aromatic ring (e.g., angelicin), whereas in the former, the same ring is attached to the 7,8 positions (e.g., psoralen). In higher plants, the most abundant linear furanocoumarins are psoralen, xanthotoxin, bergapten, and isopimpinellin, while the most relevant angular furanocoumarins are angelicin, pimpinellin, sphondin, and isobergapten (Figure 1).

Coumarins are lactones which can open on treatment with a base to *cis*-*o*-hydroxycinnamic acids and spontaneously cyclize again on acidification. Irradiation of the *cis*-cinnamate causes *cis-trans* isomerization and lactonisation. It is evident then that coumarins derive from shikimic acid via cinnamic acids; organisms possessing an enzyme system capable of *o*-hydroxylating cinnamate are thus able to biosynthesize them.

**Scheme 1:**
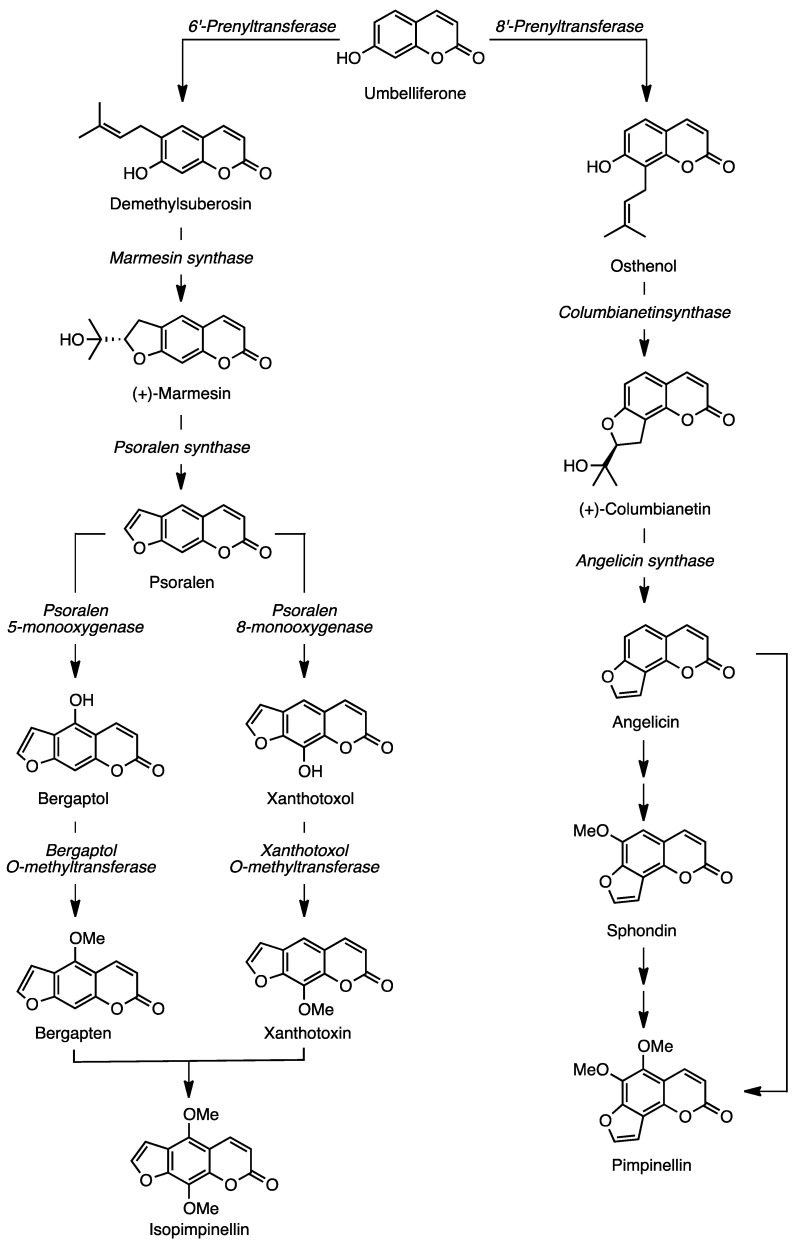
Biosynthetic pathways to linear and angular furanocoumarins starting from umbelliferone.

In detail, their biosynthesis proceeds from umbelliferone through the mevalonate pathway [1]. In plants, umbelliferone (7-hydroxycoumarin, Scheme 1) is considered as the precursor of both linear and angular furanocoumarins. Umbelliferone is a phenylpropanoid and it is synthesized from l-phenylalanine, which in turn is produced via the shikimate pathway. The biosynthesis of both linear and angular coumarins proceeds via the prenylation either at the 6-position for linear psoralens, or at the 8-position for angular angelicins. The enzymes, 6′-prenyltranferase and 8′-prenyltranferase, using dimethylallyldiphosphate as the alkylating agent, produce demethylsuberosin and osthenol, respectively [2]. Demethylsuberosin is then converted into (+)-marmesin by marmesin synthase. Subsequently, psoralen synthase catalyzes the aromatization of the dihydrofuran ring by the elimination of an acetone molecule (and one hydrogen atom) [3]. Psoralen 5-monooxygenase and the analogous 8-monooxygenase oxidize the respective positions to produce bergaptol and xanthotoxol, which are methylated each by their specific *O*-methyltransferase [4,5]. The formation of isopimpinellin, i.e., 5,8-dimethoxypsoralen, has not been elucidated yet [6,7]. 

As for the angular derivatives, less is known than for the linear ones. Osthenol is converted by the enzyme, columbianetin synthase, into (+)-columbianetin, which is in turn transformed into angelicin by an angelicin synthase [8]. The enzymes for the synthesis of sphondin and pimpinellin have not been isolated to date.

### 1.1. Chemical Diversity in Natural Furanocoumarins

More than 50 natural furanocoumarins are known, and several screenings have revealed a consistent qualitative and quantitative heterogeneity at both intra-generic and intra-specific level [9]. As a result, a large variability in biological activities has been observed for plants containing furanocoumarins and different degrees of toxicity and drug interactions may be encountered in those used as foods [10]. The literature reports a wide range of concentrations, which can be explained by a combination of natural and genetic factors, influence of storage or processing, and by constraints at both sample treatment and analytical protocols [11]. Unfortunately, pharmacological studies have rarely provided an adequate chemical characterization of the plant material and, at the same time, the evaluation of the dietary intake for furanocoumarins is considered extremely difficult [12]. On this regard, data on average human consumption from food plants are deemed scarcely reliable and actual exposure could exceed published estimates by an order of magnitude [13].

This scenario can be explained by the peculiar nature of these substances, whose histological accumulation and distribution may differ from those usually found for other secondary metabolites. Furanocoumarins represent in fact a striking example of evolution-driven phytochemical diversity, whose peculiarity have relapses on the definition of the optimal protocols for the analytical determination of their content in plants and food.

From a chemotaxonomical standpoint, furanocoumarins have been isolated from few genera within a limited group of plant families, including Apiaceae, Fabaceae, Moraceae, and Rutaceae and, to a minor degree, Amaranthaceae, Asteraceae, Cyperaceae, Meliaceae, Pittosporaceae, Rosaceae and Solanaceae [14,15]. Apiaceae and Rutaceae provide the larger number of species and the most valuable sources of furanocoumarins for pharmacological and toxicological purposes. In terms of food and medicinal plants, Apiaceae usually harbor, on a fresh-weight basis, a higher content than Rutaceae [9].

A unique pattern describes the botanical distribution of linear and angular furanocoumarins, whose emergence in phylogenetically unrelated taxa suggests an exemplary case of convergent evolution in response to specific environmental stressors. As angular furanocoumarins have been reported only in some members of Fabaceae, Moraceae, and Apiaceae, researchers have demonstrated that the emergence of these compounds was the result of the evolutionary pressure exerted by the development of detoxification pathways against linear furanocoumarins in some predators [16]. This evidence supports the hypothesis that angular furanocoumarins have evolved more recently, and this is confirmed by the absence of Apiaceae members capable of producing angular molecules only, while some species (e.g., *Petroselinum crispum* (Mill.) Fuss) synthesize only linear furanocoumarins and others (e.g., *Pastinaca sativa* L.) produce both linear and angular furanocoumarins.

In nature, the detoxification process of furanocoumarins in insects is known to involve a rapid cytochrome P450-mediated metabolism, with CYP6B enzymes particularly efficient and specific for furanocoumarins metabolization [17]. On this regard, the natural capability of some furanocoumarins to interact, inactivate, and influence microsomal enzymes is the foundation of their relevance in drug–herb interactions and of their role in the modulation of pharmacokinetic properties of multiple substances in vivo [18].

In plants, furanocoumarins play a key role as phytoalexins and they are known for their preeminent involvement in both constitutive and induced plant defense, acting both against microorganisms, nematodes, phytophagous insects, herbivores, and plant competitors [19,20,21]. The biosynthesis of furanocoumarins can therefore be induced and enhanced by direct exposure to microorganisms, insects, and fungi, as well as by abiotic elicitors, such as UV radiation and physical damage, with different intensities resulting in different accumulation ranges. Their toxicity in nature is based on direct contact and photoactivation and it is dependent on their ability to create DNA adducts under the influence of UV-A, giving cross-links in DNA and ultimately resulting in a potent cytotoxicity and acute inflammation in animals, making furanocoumarin-containing plants a class characterized by high bioactive and toxic potential [8]. On this regard, different toxicities are described for linear and angular furanocoumarins, such as psoralene-type linear furanocoumarins, like xanthotoxin and bergapten, which show strong photosensitizing effects in contrast to angelicin-type angular forms, whose phototoxic effect is weaker.

### 1.2. Localization in Tissues and Organs and Consequences for Sampling

Given the defensive role and the mechanism based on direct contact, most plants accumulate furanocoumarins prevalently on their epidermis. In particular, both linear and angular furanocoumarins are mostly extruded from epidermal cells and migrate through the waxy cuticular layer, producing crystals on the outermost side of the epidermis [22,23]. 

Both scanning electron and light microscope observations have revealed the presence of a solid furanocoumarin layer covering the cuticle of different plant organs, whose thickness is dependent on the developmental stage [23]. It has been demonstrated that its magnitude is higher in younger leaves, while during leaf expansion it may be stretched and crumbled, leading to a physical loss. This difference with other phytochemicals, usually segregated in vacuoles or in dedicated tissues, explains why a careful sample handling is needed to obtain reliable quantitative results in furanocoumarin determination. Up to 50% of total furanocoumarins in *Ruta graveolens* L., for instance, has been found to be accumulated on the leaf surface and techniques involving brief dipping of leaves into hot water or in pyridine allowed an increase in the removal of furanocoumarins from one to three orders of magnitude if compared to conventional extraction with organic solvents at room temperature [24]. Similar approaches have led to a rather easy clean up and pre-concentration of the samples for analytical purposes, but, at the same time, this evidence suggests that an incomplete extraction may be obtained whenever this peculiarity is not properly considered. For instance, mechanical solicitations during storage, freezing, grinding, and previous washing of the plant material for cleaning purposes may induce alterations of the outer layer rich in furocoumarin crystals, thus influencing quantitative results. Whenever suitable, a preferential recourse to fresh plant materials may be therefore suggested to avoid losses.

This behavior is not only a consequence of histological organization, since in vitro grown cell cultures may also harbor more than 60% of their furanocoumarins content on the surface of their cell walls instead of in their protoplast [25]. At the same time, the phytochemical profile may be variable according to the organization level in cultures, as dispersed cells were less productive than aggregated and organized calli, in which separated sites for the biosynthesis of distinct furanocoumarins have been found. This suggests that a differential biosynthetic capability may be acquired by plant cells during differentiation, establishing a link to various studies highlighting an organ-specific accumulation for furanocoumarins [26]. Furthermore, furanocoumarins are not translocated in the phloem, thus confirming the coincidence between localized biosynthesis and accumulation in specific organs or tissues [27].

As in many other secondary metabolites, an uneven distribution within a given plant has been described for furanocoumarins. As a rule of thumb, higher contents may be found in epigean and photosynthetically active parts, while the concentrations in fruit and roots are usually markedly lower. However, relevant differences may be observed even within a single organ or apparatus. For instance, when different members of the Apiaceae family have been evaluated, the presence of furanocoumarins on the fruit surface was combined with a differential accumulation in internal tissues. In fruit and seeds of *Angelica archangelica* L., furanocoumarins were present only in low concentration on the fruit surface and their total amount was inversely correlated with fruit size, with flatter seeds containing 35 times the concentrations found in turgid seeds, suggesting an inverse correlation between furanocoumarin concentration and size or maturity of the embryo [28]. The key role of embryos in the total abundance of fruit and seeds has been confirmed many times in various species, and also seeds without this structure have very low concentrations [29,30,31]. As *A. archangelica* is also known for its phototoxic properties, an uneven accumulation of furanocoumarins in different organs may result in contact dermatitis of variable intensity.

Species-specific profiles may also occur, such as in *R. graveolens* shoots, where linear furanocoumarins are present in higher amounts in the epidermis, with lesser amounts in parenchymas, and only traces in the internal secretory glands [32]. In most occasions, the accumulation is, however, not exclusively epidermal and variable amounts may be detected also in internal parenchymas or secretory tissues, as confirmed by histological investigations of *Heracleum lanatum* Michx. and *H. mantegazzianum* Sommier & Levier fruit canals. The *Citrus* genus revealed a precise distribution, with the envelope of the fruit being the richest part in terms of both molecular diversity and compound concentration [33,34]. Separate analysis of the seed coat and embryo has shown that a substantial deposit of furanocoumarins is embedded in the wax present in the overcuticular layer of the embryos, thus repeating a pattern already evidenced in leaves and shoots [22]. 

Besides the embryo, a structure known for its elective accumulation of furanocoumarins is the petiole. In *Apium graveolens* L., psoralen and isopimpinellin are dominant in laminas, while bergapten is dominant in petioles. Cross sections of petioles of unexpanded leaves, examined by fluorescence microscopy, have revealed the presence of furanocoumarins in oil channels associated with each vascular bundle [35]. Similarly, the biosynthetic pathways in *Ficus carica* L. seem to be closely located in the epidermal cells of leaf nerves and in the petiole, in which linear furanocoumarins, such as psoralen, bergapten, and xanthotoxol, both in free and glucosylated form, were found in higher quantities if compared to leaf blades, thus revealing higher concentrations in the abaxial side of the leaf [36]. 

This specific accumulation implies that peeling vegetables may result in a significant reduction of furanocoumarin content and, therefore, the analytical determination in food or in medicinal plants should carefully describe the handling of the plant material. Furthermore, an analysis aimed at the determination of the dietary exposure to these compounds should consider the consequences of household processing usually performed to prepare food. It has been observed that *Citrus* fruit peel does not simply provide a higher content of total furanocoumarins, but it also has a broader diversity if compared to the corresponding pulp. The differential distribution also has relapses when processing is involved, as in the case of food. For instance, *Citrus* × *paradisi* Macfad. juice processed by means of blending may provide higher concentrations of bergamottin, while hand-squeezing results in higher concentrations of 6,7-dihydroxybergamottin if compared to commercially processed juice [37]. In processed *Citrus* juices, the relative concentrations of furanocoumarins have been reported to be different between the raw finished juice, centrifugal retentate, centrifuged supernatant, and coarse finisher pulp, in which centrifugal retentate provided the highest furanocoumarin content [38].

Overall, these evidences suggest that a careful description of the plant material used for pharmacological and phytochemical investigation should be provided and that more potent or concentrated samples may be obtained by using specific plant tissues as the starting point. 

### 1.3. Factors Affecting Furanocoumarin Content in Plants

As in many other plant secondary metabolites, furanocoumarin biosynthesis may be modulated by the developmental stage of plants and seasonality. A higher percentage has been generally observed on the surface of younger compared to mature organs, in particular in leaves, thus evidencing a net seasonal effect with values that for xanthotoxin, bergapten, and psoralen in *H. lanatum* may show a 20 to 100 times higher furanocoumarin content during the new growth occurring in autumn. The developmental stage of the leaves affects the degree of accumulation, with leaf enlargement being inversely correlated with furanocoumarin richness and, similarly, the content of bergapten in petioles of *A. graveolens* declines from early developmental stages through to maturity [39,40]. In *R. graveolens* and in other plants, this behavior extends to immature seeds, which present a higher furanocoumarin accumulation on their surface if compared to mature ones. Year-to-year variability has been reported to exceed 190%, at least in *P. sativa,* in which temperature and stress have been found to play a crucial role. The medicinal and toxic plant *Angelica dahurica* (Hoffm.) Benth. and Hook.f. provides a good example of the relevance of the collecting time on the phytochemical profile of furanocoumarins. Indeed, its taproot and lateral roots have shown different patterns of furanocoumarins, according to their harvest stage, with different compounds providing yields variable up to 50% at different harvest stages. As a rule of thumb, when below-ground are to be collected, a general suggestion seems to be focused on the summer dormancy stage as the best harvest period, because of its richer total furanocoumarin content [41]. To maximize furanocoumarins’ yield, the optimal collection time for aerial parts is therefore represented by early spring and any sampling for analytical purpose should precisely describe the time of collection for the plant material.

Furanocoumarins are stress-induced compounds; thus, their abundance is also a consequence of the exposure to precise conditions and mechanical solicitations, which may influence both the biosynthesis and the accumulation. Various elicitors of furanocoumarin biosynthesis have been described, with temperature and mechanical damage exerting the most relevant effects. Being part of the inducible defense system, the levels of these metabolites may in fact increase because of an exposure to pathogenic fungi or to physical damage caused by occasional lesions or insect bites, as confirmed by experiments performed both in vitro and in vivo [40]. For instance, cultured cells under vigorous shaking produced amounts of furanocoumarins up to two orders of magnitude greater than calli [25]. In *P. sativa* leaves, both insect bites and manual damage have induced furanocoumarin production with increases for single compounds ranging from 140% to 270%. Manually inflicted damage has produced a less remarkable but still significant induction. This confirms the role of furanocoumarins, but also highlights the potential difference from analytical results obtained from fresh, entire leaves, and material exposed to mechanical solicitations, including pre- and post-harvest handling, suggesting once again that sample treatment may represent a key factor in assuring repeatability and reliability of analytical evaluations. It has been observed that levels of furanocoumarins in non-damaged samples of transported and stored plants are lower if compared to plants mechanically injured or rotten during transportation, where up to a 10 times higher content has been detected [11,42]. Therefore, the period between the sampling and the analysis should be minimized and the potential difference between content in fresh healthy plant foods and even slightly damaged ones should be considered when analyses are performed to determine dietary exposure of furanocoumarins.

Environmental factors, such as the exposure to air and water pollution, are also known to stimulate furanocoumarin biosynthesis, as reported for *A. graveolens* exposed to an acidic fog, in which the concentration of psoralen, bergapten, and xanthotoxin increased up to 500% with respect to the controls, while no influence was reported for increased CO_2_ availability and exposure to pesticides [33,43]. On the contrary, bergapten content in *A. graveolens* has increased up to 2 to 4 times in leaves and stalk; xanthotoxin increased 2 to 3 times in stalk, and isopimpinellin about 2 to 3 times in leaves when treated with fungicides [44]. The influence of the phenotypic diversity and the intraspecific chemodiversity have also been evaluated in adequate depth only for *Citrus*, suggesting that plants related to *Citrus maxima* (Burm.) Merr., *Citrus lemon* L., *Citrus micrantha*, and *Citrus hystrix* may accumulate these substances in high amounts, whereas *C. deliciosa* related species (including most common edible agrumes, like *Citrus aurantifolium* and *Citrus sinensis*) appear almost devoid of them. Regarding hybrids, their corresponding chemotypes appear inherited from respective ancestral taxa, with a prevalence of *C. maxima*, *C. lemon*, *C. micrantha*, and *C. hystrix* related species and hybrids [45]. Similar studies are not available for other plants, such as those used for medicinal purposes; thus, a suitable investigation in this context may be advisable.

## 2. Extraction of Furanocoumarins from Plants

Extraction represents a crucial step for both the development of an analytical method for the study of the composition of a plant material and for the isolation of natural compounds [44]. The extraction protocol applied can vary according to the property of both the plant material and the molecules of interest. Many factors can substantially affect the extraction yield, including the extraction technique, the solvent type, and so on [46]. 

Based on what has been previously mentioned on the distribution of furanocoumarins in plant tissues, the sample pre-treatment should be as simple as possible. Grinding, freezing, and washing processes should be avoided, especially when it comes to work with leaves [24]. A simple dipping of the fresh plant material in boiling water, followed by liquid-liquid extraction (LLE) with ethylacetate (EtOAc) and further partitioning with organic solvents has been applied by Zobel et al. as the best extraction technique for a reliable quantification of furanocoumarins from *R. graveolens* leaves [24]. As a consequence, sample pre-treatments, including grinding and freezing of fresh plant material, and conservation for a long time of dried plant specimens as well, may result in physical losses of waxy cuticular material, therefore affecting the final quantification of furanocoumarins [47,48,49,50,51,52,53,54,55,56,57,58,59,60]. 

In general, polar solvents, such as water (H_2_O), methanol (MeOH), ethanol (EtOH), acetone, and their aqueous mixtures, are the most widely used as extraction solvents [47,49,50,51,52,53,54,55,57,58,60,61,62,63,64,65,66,67,68,69,70,71,72,73]. The use of EtOAc, chloroform, dichloromethane, and petroleum ether has also been described in the literature [56,59,61,67,74,75,76,77,78]. Kvievis et al. have recently evaluated the content of furanocoumarins in the seeds of *P. sativa* [79]. The authors prepared a raw extract by means of a simple maceration of the fresh seeds with pyridine, which has been partitioned with solvents with different polarity [79]. The extraction efficiency of different solvents has been compared also by Peroutka et al. on furanocoumarins from the peel and pulp of *Citrus* fruits, finding MeOH as the optimum solvent for the extraction of this class of compounds [9]. 

An innovative approach for the extraction of furanocoumarins from *F. carica* leaves has been recently applied by Wang et al., who have used deep eutectic solvents (DESs), which usually combine a few components capable of associating by hydrogen bond interactions to form an eutectic mixture [80]. Negligible volatility, non-flammability, favorable thermal and chemical stabilities, and strong solvation ability for most compounds represent the main advantages of using these “green” solvents [80]. In particular, Wang et al. concluded that tailor-made DES composed of glycerol, xylitol, and d-(−)-fructose (Gly:Xyl:Fru) provides much higher extraction yields when compared with MeOH; the best results have been obtained by combining DESs with microwave-assisted extraction (MAE) [80].

Several solvents have often been employed for the extraction of furanocoumarins for the analytical characterization of *Citrus* species juice by means of LLE. In this case, the necessity to form two separate layers limits the choice of the extracting solvent, with the reciprocal immiscibility with the aqueous juice being a mandatory prerequisite. Thus, highly polar solvents, such as MeOH, EtOH, and acetone, are not suitable for LLE [46]. Gattuso et al. have selected dimethyl-formamide (DMF) as the most suitable solvent for the extraction of the target compounds from *Citrus bergamia* Risso [81]. Vander Molen et al., working on *C.* × *paradisi* juice, have used EtOAc for the extraction of furanocoumarins and flavonoids, *C.* × *paradisi*, and they have developed a rapid quantitative method [82].

Solid-liquid extraction (SLE) with organic solvents by means of conventional extraction techniques still represents the most frequently used technique for the extraction of furanocoumarins from different plant material. In this ambit, static and dynamic maceration at room temperature from 30 min to 3 days have been used for both the extraction of furocoumarins for analytical purpose and for their isolation from different plant material, even if to a lesser extent [47,49,57,58,59,63,64,67,68,69,73,76,78,79]. Soxhlet extraction has been rarely applied in the ambit of the qualitative and quantitative analysis of furanocoumarins from different plant sources, since it represents a time and solvent consuming process, although it allows for an exhaustive extraction of the target compounds. Nevertheless, Shinde et al. selected an extraction protocol involving the use of a Soxhlet apparatus at 70 °C for 3 h [53]. Regarding more efficient techniques, Waksmundzka-Hajnos et al. tested different approaches for the extraction of furanocoumarins from *Archangelica officinalis* Hoffm. fruits [48], including Soxhlet extraction, ultrasound-assisted extraction (UAE) at 25 and 60 °C, MAE in open and closed systems, and accelerated solvent extraction (ASE). The highest yield of furanocoumarins was obtained by ASE with MeOH at 100 to 130 °C for 10 min, while the extraction yields of furanocoumarins from plant material by UAE and MAE in an open system were not different to those obtained by a Soxhlet apparatus [48]. Despite these findings, UAE represents the technique of choice for sample preparation in the ambit the analysis of furanocoumarins in the extracts of different plant material, probably due to its feasibility in terms of fastness and cost, in addition to its efficacy [50,51,52,54,55,56,60,66]. In most cases, UAE has been carried out at room temperature, with the exception of Zhou et al., who applied a temperature of 30 °C [60]. For what concerns the time of the UAE cycles, it ranges from 20 to 40 min [50,51,52,54,55,56,60,66].

MAE represents another extraction technique widely diffused in the ambit of the analysis of natural products. It should be pointed out that Waksmundzka-Hajnos et al. observed that MAE in a closed system can cause a degradation of furanocoumarins, ascribable to microwaves [48]. Nevertheless, this technique has been described in the literature for the extraction of furanocoumarins [52,65,80]. The time of exposure of the extraction mixture to the microwaves ranged from 1 to 44 min, while the optimal extraction temperature has been found to be 50 °C [52,65,80].

Supercritical-fluid extraction (SFE) with CO_2_ represents a good alternative to the above mentioned SLE techniques. Indeed, linear furocoumarins have been extracted from fresh *A. graveolens* var. *rapeceum* by using SFE [62]. The SFE method has been optimized by comparing the extraction yield at different CO_2_ densities, with the optimum recovery of furocoumarins obtained when the density was 0.58 g/mL. The yields by SFE were in agreement with those obtained from Soxhlet extraction [62]. The green SFE approach has been adopted also by Santana et al., who applied it to the evaluation of the furanocoumarin content in young and adult leaves of *Zanthoxylum tingoassuiba* A. St.-Hil. [83].

Prior to HPLC analysis, solid-phase extraction (SPE) has been often used for sample clean-up [84,85,86,87]. The extraction recoveries of aqueous bergamottin and bergapten from *C.* × *paradisi* have been compared by different SPE sorbents by Prosen et al. [86]: A reversed-phase DSC-18LT cartridge provided the best results in terms of extraction recovery. Zgórka et al., after the extraction of furanocoumarins by means of reflux extraction with MeOH, carried out a further purification step by using octadecyl (C_18_) microcolumns [84]. Normal phase sorbents can also provide good results as a consequence of the semi-polar behavior of furanocoumarins. Indeed, Ostertag et al. and Messer et al., after SLE or LLE of the plant material or *C.* × *paradisi* juice, respectively, submitted the organic phase to a first SPE purification step with a C_18_ column and the eluate was then further purified under normal phase SPE with a silica column [85,88].

In recent years, the innovative Quick, Easy, Cheap, Effective, Rugged, and Safe (QuEChERS) approach has been applied for the extraction of furanocoumarins from *C.* × *paradisi* juice and food for analytical purposes [14,89,90]. After homogenization of the sample (in the case of food), acetonitrile (ACN) is added together with the QuEChERS powder, consisting of 4 g of magnesium sulfate and 1 g of sodium acetate and the internal standard. After mixing for 1 to 5 min, the organic layer is collected and submitted directly to HPLC analysis [14,89] or to a purification step on a dispersive solid phase extraction (dSPE) tube [90].

If the final purpose is the isolation of furocoumarins from the plant material for the evaluation of their biological activity, the raw extract obtained has been usually submitted to further purification steps [52,65,66,69,70,71,72,74,91]. In this case, Soxhlet extraction and heat reflux extraction (HRE) have been widely applied with both polar and non-polar solvents [71,72,74,75,77]. After the extraction, furanocoumarins have been frequently isolated from the raw extracts by means of sequential silica gel column chromatography, eluted under normal phase conditions [66,69,70,71,72,75,76,77,78,79]. It is worth noting that preparative counter current chromatography has also been applied to achieve the final purification of furanocoumarins from *Angelica dahurica* and *Toddalia asiatica* (L.) Lam. roots [52,65].

## 3. Analysis of Furanocoumarins in Plants

Several analytical strategies have been employed for the identification and qualitative-quantitative analysis of furanocoumarins in plants, derived food products, and cosmetics. Historically, thin layer chromatography (TLC) has been exploited to identify different types of compounds [92]; however, with the technological progress of instrumental analysis, strategies based on liquid chromatography (HPLC) and gas chromatography (GC) have proven to be an irreplaceable tool for phytochemical profiling and for the definition of furanocoumarin content in plant samples.

In particular, a careful investigation of the scientific literature on the subject shows how HPLC coupled to different detection systems is the most exploited analytical strategy for this purpose. As far as the identification of new compounds and qualitative profiling is concerned, the most frequently applied detection is represented by medium and high resolution mass spectrometry (MS, MS/MS, and HRMS) [38,50,58,59,70,73,76,79,81,84,85,86,87,88,89,90,91,92,93,94,95,96], while for quantitative targeted purposes, other detection systems have proved to be useful, such as ultraviolet (UV), diode array (DAD) [38,53,67,77,81,88,90,97], and fluorescence detection (FLD) [89,98,99,100]. The high selectivity provided by MS is essential for isomeric compounds, such as bergapten and xanthotoxins. In this ambit, Li et al. have investigated the MS fragmentation of several linear furanocoumarins from an *A. dahurica* root extract and they have been able to separate them according to their different fragmentation pattern [51].

In general, research work taking into consideration targeted analysis aim at studying furanocoumarin level variations (alone or together with compounds belonging to other classes), with the variation of production processes and storage conditions [67,85], plant cultivars [57,81], harvest year [79], composition variability of commercial products [88], or as quality control of the raw material and commercial products [53,58]. Often, instrumental analysis is combined with other assays, e.g., antioxidant activity evaluation [67,77,81], biological assays [73,77], pharmacokinetic studies [89,94], and other instrumental techniques, aimed at compound identification, e.g., nuclear magnetic resonance spectroscopy (NMR) [59,70,76,79,81,95] and Fourier transform infrared spectroscopy (FTIR) [75].

Moreover, it is possible to find in the literature some advanced and promising analytical approaches worth mentioning: Chen et al. carried out a direct analysis in real-time mass spectrometry (DART-MS) coupled to ion trap mass spectrometry for the profiling of traditional Chinese medicines (TCMs) (*Fraxini* cortex, *Angelica pubescens* Maxim. radix, *Peucedani radix*, and *Psoraleae fructus*), containing different phytochemicals, including simple coumarins, furanocoumarins, and pyranocoumarins [56]. 

Cook et al. presented the analysis of a dataset from comprehensive two-dimensional liquid chromatography (LC × LC) coupled to DAD and applied to the analysis of *P. crispum*, *P. sativa* and *A. graveolens* samples for the quali-quantitative determination of 14 furanocoumarins. Moreover, a chemometric approach represented by multivariate curve resolution-alternating least squares (MCR-ALS) has been successfully applied to isolate pure analyte signals from the background ones and to resolve overlapping peaks [90].

A chromatographic fingerprint analysis and characterization has been applied to identify or tentatively characterize a total of 20 furanocoumarins from the roots of *A. dahurica* by investigating MS/MS fragmentation patterns of the furanocoumarins in an electrospray ion trap mass spectrometer (HPLC-DAD-ESI-MS^n^) [50].

Photoactive furanocoumarins (bergaptol, psoralen, 8-methoxypsoralen, bergapten, 6′,7′-dihydroxybergamottin, epoxybergamottin, and bergamottin) have been analyzed in *C.* × *paradisi* parts (whole, flesh, peel, and juice) by using ultra-high-performance liquid chromatography coupled to mass spectrometry (UHPLC-MS/MS). Moreover, furanocoumarin levels in plasma and urine samples obtained from six healthy volunteers before and after consumption of an entire fruit or juice of the same species have also been assessed to investigate their bioavailability [89]. 

A study aimed at characterizing and discriminating 44 commercial samples of essential oils isolated from different *Citrus* species (*C. bergamia*, *C. limon*, *C. aurantium*, *C. sinensis*, *C. deliciosa*, *C.* × *paradisi*, *C. aurantifolia*) by analyzing the non-volatile oxygenated heterocyclic compounds by UHPLC and HRMS has also been described, by using multivariate statistical analysis and metabolomic strategies. The analyzed fraction included coumarins, furanocoumarins, and polymethoxylated flavonoids. A targeted profiling based on the quantitation of 18 furanocoumarins and coumarins and a targeted fingerprinting based on a database of 140 compounds already reported in *Citrus* essential oils have been used, from which 38 discriminant markers have been defined to discriminate the *Citrus* species and assess a possible adulteration [68].

The essential oil composition and furanocoumarin profiles of underground parts and fruit extracts obtained from *Heracleum* taxa have been statistically analyzed to evaluate their chemosystematic significance. Furanocoumarins have been identified by using standards and/or based on UV, MS, and NMR spectra, while qualitative and quantitative analysis has been performed by HPLC-MS. Multivariate statistic of analyzed metabolites has shown that the investigated taxa can be classified according to their taxonomy. PCA has revealed the significance of most of the 12 identified furanocoumarins [59].

Finally, a few papers describe a novel chromatographic approach, based on supercritical fluid chromatography (SFC), exploited for the effective separation and analysis of several furanocoumarins in the roots of *A. dahurica* [55] and a *C. limon* fruit residue [101]. After a careful investigation regarding the main parameters involved in the chromatographic resolution of compounds, the results have shown how SFC could represent an efficient, “green,” and practically relevant separation technique with several unique features, as a very limited production of solvent waste. Consequently, it may be considered as a promising alternative for furanocoumarin analysis.

## 4. Synthesis of Furanocoumarins

Besides their extraction from plants, furanocoumarins may be synthetized for pharmaceutical purposes with different approaches, depending on which of the three rings are built in the cyclization step. In practice, the different furanocoumarins can be obtained either by the construction of a furan ring on a coumarin, formation of the pyrone on a benzofuran, or by synthesis of both the furan ring and the pyron ring around a central benzene. The topic has been extensively reviewed elsewhere [4].

**Figure 2:**
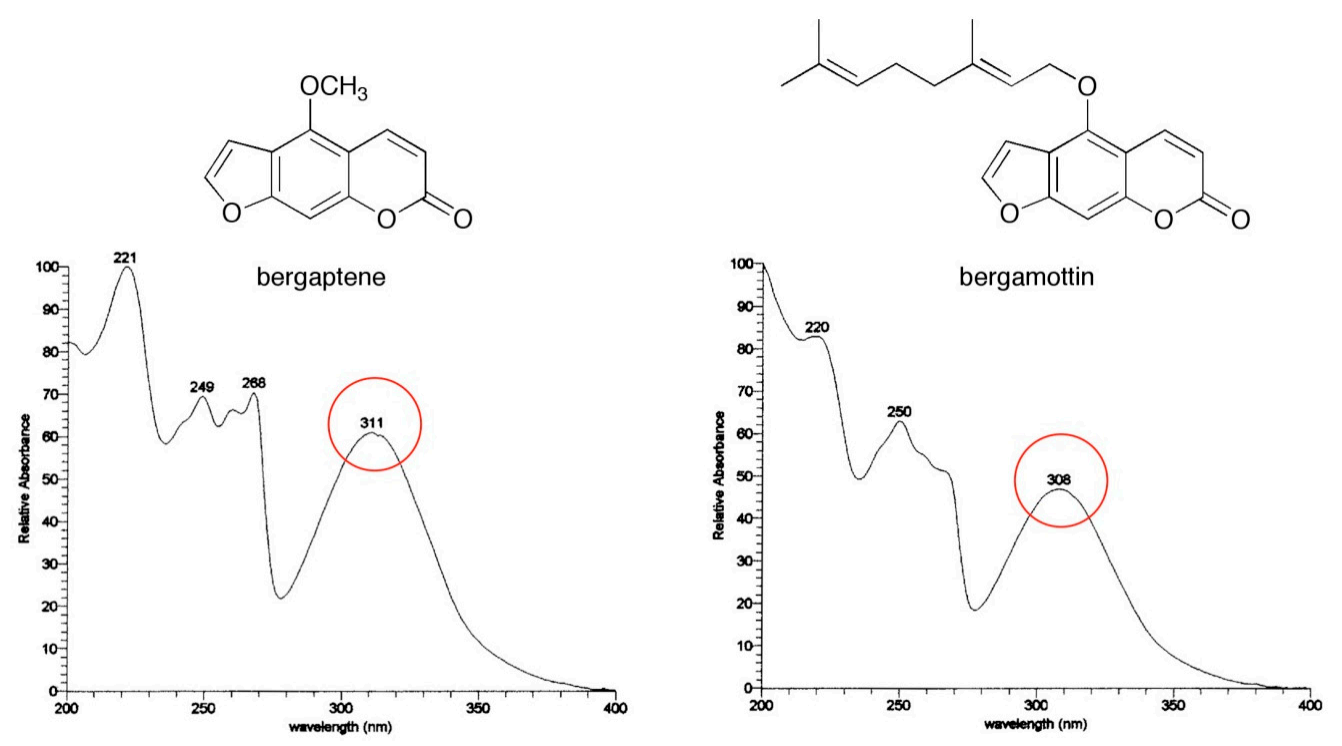
UV/Vis spectra of bergaptene (**left**) and bergamottin (**right**), two psoralens from *Citrus bergamia* Risso.

Psoralens absorb UVA light and they are photosensitizers in the 320 to 380 nm range [5], where nucleic acids and proteins do not absorb significantly. The UV spectra of two sample psoralens are depicted in Figure 2. Their photochemical activity is determined by the nature of the lowest excited states (S_1_ and T_1_ π–π* states). 

The photochemical and photophysical behaviors of furanocoumarins in water have been investigated in depth, with the aim of shedding light on their photobiological properties at the molecular level. The initial formation of furanocoumarin molecular complexes with DNA involves weak forces (such as Van der Waal’s attractions and hydrogen bonding). After irradiation, psoralens bind to DNA by means of [2+2] photocycloaddition reactions with pyrimidine nucleobases (e.g., thymine). 

On the basis of the results obtained in the areas of molecular photochemistry, photobiology, and X-ray crystallography, furanocoumarins have found application both as drugs and as molecular probes.

## 5. Biological Activities of Furanocoumarins

Furanocoumarins have shown many interesting biological activities both in vitro and in vivo, such as antioxidant, anti-inflammatory, antiproliferative, and as promoters of bone health, making them the focus of a great deal of investigations for both academic and industrial researchers. Furocoumarins effects on living organisms are complex and many questions remain open, especially as far as the safety of their use in medical therapies and consumption through the diet [102]. In particular, linear furanocoumarins have been historically employed in the treatment of skin disorders, such as to stimulate pigmentation in vitiligo, psoriasis, and leukoderma, polymorphous dermatitis, eczema, and mycosis, but the toxicity of plants rich in these compounds is also a matter of concern [103]. *A. dahurica* at high doses, for instance, has been associated to emesis, severe seizures, and paralysis in mammals, including humans [104]. It is also worth mentioning the strong anti-proliferative activity against cancer cells’ growth by modulating different biochemical pathways, such as the regulation of mitogen-activated protein kinase expression, signal transducers, and activators of transcription 3, phosphatidylinositol-3-kinase/AKT and nuclear factor-kB. Moreover, some furanocoumarins and, in particular, some fruit and juice, as those obtained from *C.* × *paradisi*, are responsible for adverse effects at the therapeutic dose of administered drugs, due to their interaction with cytochrome P450 isoforms, multidrug resistance protein 1 (MDR1), organic anion transporting polypeptides (OATP), and P-glycoprotein [105,106,107]. Linear furanocoumarins exert photosensitization towards UV light, resulting in sunburn or serious blistering in humans, and they are employed in medicinal applications to favor skin pigmentation and the treatment of psoriasis. If not properly treated, essential oils obtained from the peel of the fruits of *C. bergamia* are rich in bergapten and bergamottin, and it has been extensively used in external suntan preparations, absorbing light in the near UV region and stimulating the formation of melanin [98]. On this regard, careful use must be advised given the proclivity of these furanocoumarins to induce strong cellular damage and harsh skin inflammation after photoactivation. After absorption of a photon, they form a triplet excited state that reacts with pyrimidine bases (as mentioned above, forming monoadducts and/or cross-linking adducts able to induce cytoplasmatic mutations) or with oxygen (forming reactive oxygen species able to damage all biological macromolecules, such as protein, DNA, RNA, lipids, and carbohydrates).

The DNA binding potential, interfering with the basic biological functions of this macromolecule, is one of the main causes of their toxicity towards a wide spectrum of organisms, including mammals, viruses, bacteria, plants, insects, and fungi [14]. On the other hand, their ability to interact with and disrupt DNA replication may represent a starting point to develop anti-cancer agents and therapies based on furanocoumarins [108]. Several in vitro studies have analyzed the inhibitory effects of furanocoumarins on the growth of various cancer cell lines, including breast cancer and non-small cell lung cancer [109,110,111], with psoralen representing a compound commonly employed for the treatment of cutaneous T cell lymphoma in extracorporeal photophoresis, where malignant cells are irradiated with UVA and then reinfused into the subject undergoing the treatment [112]. In recent studies, bergamottin, bergaptol, and bergapten have shown inhibitory effects on breast cancer cell growth. Bergamottin treatment of MDA-MB-231 breast cancer proliferation has caused a significant reduction of phosphorylation, nuclear translocation, expression, and DNA binding activity of signal transducers and activator of transcription 3 (STAT3) [113]. When MDA-MB-231 cells have been exposed to bergamottin (100 µM for 6 h), two different phenomena have been observed: A reduction of phosphorylation, a nuclear translocation of STAT3, and a suppression of binding activity of the STAT3 protein to the corresponding DNA sequence. The STAT3 signaling pathway is often activated in malignant cells, and it has a key role in regulating several genes crucial for cancer inflammation. Bergamottin has shown promising inhibitory activity against multiple myeloma cell growth [113]. In a time-dependent manner, it has decreased the proliferation of U266 cells by inactivation of the phosphorylation of Janus-activated kinase and c-Src protein, followed by inhibition of STAT3 phosphorylation and nuclear translocation. This last event results in the suppression of cyclooxygenase-2, vascular endothelial growth factor, antiapoptotic (such as Bcl-2, Bcl-xl, IAP-1), and survival genes. The effects of bergamottin were also evident in a synergistic combination with simvastatin, enhancing its anticancer effects against human chronic myelogenous leukemia. The treatment of the cells with these last two molecules significantly potentiated the inactivation of nuclear factor kB (NF-kB) expression and the induction of apoptosis. Moreover, the effects on NF-kB resulted in a further downregulation of Bcl-2, Bcl-xL, cyclin D1, VEGF expression, and matrix metallopeptidase 9 [114]. Bergamottin may also have a role in the treatment of skin cancer, gastric carcinoma, and neuroblastoma, and in the suppression of metastasis. Cai et al., in an experimental mouse skin carcinogenesis models utilizing benzo[a]pyrene as an inducer, tested five different coumarins and furanocoumarins [115]. Bergamottin demonstrated the highest potential to avoid covalent binding of benzo[a]pyrene to mouse epidermal DNA, modulating cytochrome P450 enzyme activity and, therefore, affecting metabolic activation of benzo[a]pyrene. In NCI-87 gastric carcinoma cells, bergamottin (4–100 μM for 48 h) has shown the highest activity among the 25 different potential nutraceuticals analyzed for the treatment of *Helicobacter pylori* infections, blocking CD74 expression with an IC_50_ value of 14.2 μM [116].

In a recent in vitro study, some components of *C. bergamia* essential oil, in particular bergamottin and 5-geranyloxy-7-methoxycoumarin, induced SH-SY5Y cell apoptosis [117]. These molecules increase the presence of reactive oxygen species and regulate the expression of mitogen-activated protein kinases, such as p38, p53, extracellular signal-regulated kinase (ERK1/2), Bax, and Bcl-2.

Bergapten is capable of a dose-dependent inhibition of inhibited breast cancer cell growth, through a blocking of the cell cycle in the G0/G1 phase and activation of p53 and caspases (caspase 8/caspase 9), resulting in the onset of apoptosis in MCF-7 and ZR-75 cells. These effects have been analyzed after the treatment of cell lines with furanocoumarins for 48 or 96 h, independently of the exposure to UV [118,119]. Moreover, the same furanocoumarins increased NF-Y nuclear translocation through p38 MAPK activation and impaired the PI3Kinase/AKT survival signal in hormone-dependent MCF-7 cells, even in the presence of IGF-I/E2 mitogenic factors. Bergapten has also been found to be able to induce autophagy, as well as apoptosis, in tamoxifen-resistant MCF-7, MCF-7, and ZR-75 breast cancer cells [119,120]. In tamoxifen-resistant MCF-7, surprisingly, bergapten reduced the amount of estrogen receptors through the blocking of the protein transduction process and the depletion of the same receptor trough SMAD4-mediated ubiquitination, without changes in its mRNA level. The treatment of MCF-7 and ZR-75 breast cancer cells with bergapten resulted in the expression of Beclin, a UV radiation resistance-associated gene (UVRAG), and in an enhancement of Beclin-1-regulated autophagy (AMBRA). As a result, a promotion of autophagosome generation has been observed. The same authors have described phosphatase and tensin homologue (PTEN) deletion on chromosome 10 as being mainly responsible for bergapten activity in the induction of breast cancer cell autophagy. Recently, Ge et al. described the activation of apoptosis in MCF-7 cells by bergaptol, inducing the expression of Bax and cytochrome c and the cleavage of caspase-9 and poly(ADP-ribose) [121].

Oxypeucedanin (prangolarin) has shown a remarkable cytotoxicity activity against HeLa cell line (with an IC_50_ value of 314 μg/mL), as well as other pharmacological and biological activities, such as antibacterial, antiarrhythmic, antifungal, channel blocker, antioxidant, allelopatic, and antiestrogenic activity [122]. Shalaby et al. described the antidiabetic activity of *Ducrosia anethifolia* Boiss organic extracts, characterized by the presence of eight linear furanocoumarins [123]. The blood glucose level, redox balance, liver function enzymes, total protein, lipid, and cholesterol levels were significantly normalized by organic extracts treatment in male Wister albino rats of a 20-week age (250 ± 50 g), in which diabetes was induced by streptozocin. The major isolated furanocoumarins acted in vitro in a concentration dependent manner as inhibitors of carbohydrate metabolizing enzymes (*α*-amylase, *α*-glucosidase, and *β*-galactosidase). Imperatorin and bergapten possess the highest inhibitory activity, whereas the lower inhibitory power has been observed for oxypeucedanin hydrate, psoralen, and isooxypeucedanin. Shin et al. demonstrated the potential of the furanocoumarin, byakangelicin, in the treatment of diabetic cataract [124], acting as an inhibitor of aldose reductase activity, and Hsu et al. analyzed pangelin (pabulenol) as an antihyperglycemic component isolated from the roots of *Paeonia lactiflora* Pall. [125]. This promising activity may have its explanation in the antioxidant and anti-inflammatory activity of these molecules, although their activity is, in general, lower than other secondary metabolites and vitamins [126,127,128,129,130,131]. Piao et al. analyzed the scavenging potential of 11 furanocoumarins isolated from *A. dahurica* against 2,2-diphenyl-1-picrylhydrazil (DPPH) radicals [132]. The IC_50_ values indicated that 9-hydroxy-4-methoxypsoralen and alloisoimperatorin have the highest activity (6.1 μg/mL and 9.4 μg/mL, respectively), while byakangelicin, byakangelicol, imperatorin, isoimperatorin, neobyakangelicol, oxypeucedanin, oxypeucedanin hydrate, pabulenol, and phellotorin have IC_50_ values clearly superior to 200 μg/mL, with very little or no DPPH radical-scavenging activities. Bergapten has been shown to possess a low antioxidant activity in the *β*-carotene-linoleic acid bleaching and superoxide radical scavenging assay, while its activities became significant against copper-induced low-density lipoprotein oxidation in hamsters and in the delay of rat brain lipid peroxidation [126,133]. Bergaptol exhibited free radical scavenging activities in different assays as in the 2,2′-azobis 3-ethylbenz-thiazoline-6-sulfonic acid (ABTS) and 2,2-diphenyl-1-picrylhydrazil (DPPH) test and its activity is superior to the other 23 furanocoumarins isolated from *A. dahurica* [134,135].

Pharmacological and biochemical studies have indicated that several furanocoumarins (isoimperatorin, oxypeucedanin, notopterol, and bergapten) have anti-inflammatory activity, as well as analgesic, anti-cancer, and anti-coagulant potential [135,136,137,138,139]. In 2015, Uto et al. evaluated the anti-inflammatory activities of constituents present in either aerial or root parts of *Angelica acutiloba* (Siebold and Zucc.) Kitag. on a RAW 264.7 cell model treated with lipopolysaccharide and found a modest anti-inflammatory activity of bergaptol, through the attenuation of the production of nitrite, prostaglandin E2, interleukin-6, and TNF-*α* [138]. This anti-inflammatory activity of bergaptol is due to the upregulation of heme oxygenase-1 expression, which regulates antioxidative activity. Oxypeucedanin has shown an anti-inflammatory activity similar to that of bergaptol, acting mainly on nitrite production and in the expression of inducible nitric oxide synthase on LPS-treated RAW 264.7 cells [137].

The biological potential of furanocoumarins has been revealed also at the brain level, as in the case of imperatonin. The molecular mechanism of the action of this furanocoumarin is due to the irreversible inactivation of *γ*-aminobutyric acid (GABA)-transaminase, increasing the GABA amount in the synaptic clefts of neurons and in the brain [140,141]. Recent experiments on imperatorin have highlighted the dose-dependent increase of the threshold for maximal electro-convulsions in mice, that reached the peak of the maximum anticonvulsant effect 30 min after systemic administration [142]. Moreover, imperatorin administration at sub-threshold doses have increased the anticonvulsant effects of conventional antiepileptic drugs, such as those of carbamazepine, phenytoin, and phenobarbital, but not those of valproate, against maximal electroshock-induced seizures in mice, resulting in an increase of the total brain carbamazepine concentration in the treated animals, but not the one of phenytoin and phenobarbital. The mechanism by which the co-administration of imperatorin induces these effects has not been elucidated, but it is possible that this furanocoumarin enhances the diffusion of carbamazepine through the blood–brain barrier by modulating its permeability and/or inhibiting the activity of the multi-drug resistance proteins or the P-glycoproteins [139,143].

In the past few years, few studies have reported the bone health promotion activity of furanocoumarins, especially bergapten, both in vitro and in vivo. Meng et al. in a search for compounds responsible for the proliferation-stimulating activities of crude extract of *Cnidium monnieri* (L.) Cusson on osteoblast-like UMR106 cells, found that bergapten significantly increased the cell proliferation percentage of UMR 106 cells by 24% in a dose- and time-dependent manner as compared to the reference control group [144]; moreover, it increased the activity of alkaline phosphatase (ALP), bone nodule formation, type I collagen synthesis, and bone morphogenetic protein-2 (BMP-2) gene expression [145]. By analyzing the possible marker of bergapten activity, the co-treatment with noggin, an antagonist of BMP-2, attenuated the expression of ALP, indicating that up-regulation of BMP-2 is involved in the mechanism of action of bergapten. The central role fulfilled by BMP-2 in the activity of furanocoumarins was evident also in young rats treated for 7 consecutive days with bergapten, where it was possible to note a remarkable increase in the bone volume, phosphorylation of SMAD 1/5/8, p38, and ERK, and BMP-2 expression at the level of the tibia. Bergapten modulation of key enzymes in bone health promotion is not limited to the above described proteins, but it involves also the expression of Runt-related transcription factor 2 (RUNX2), modulation of the Wnt/β-catenin pathway, and osteocalcin (noncollagenous protein secreted by osteoblasts) [146,147,148]. Oral administration of bergapten for 3 months to an ovariectomized mouse model (one of the main animal models for studying postmenopausal osteoporosis) resulted in a remarkable decrease in delaying bone loss as shown by analysis of the bone mineral density, separation, and number of trabeculas. The activity of bergapten involves also the suppression of osteoclastic cell growth, responsible for bone resorption, through activation of the apoptotic pathway in LPS-stimulated osteoblast precursor RAW 264.7 cells [149]. All together, these data may supply some experimental evidences on the modulation of bone strength in rats that regularly consume *C.* × *paradisi* fruits [150,151,152].

An overview of the compounds discussed in the section above is presented in tabular fashion, along with the properties that have been revealed so far (Table 1).

## 6. Conclusions

At first glance, after various decades of phytochemical, analytical, and pharmacological investigations on furanocoumarins, few novelties may be expected. At the same time, however, a comprehensive and critical review of the literature highlights the presence of key aspects that should be pursued to improve both our knowledge and the overall quality of the research in this ambit. From a technological standpoint, most of the data available on the distribution and abundance of furanocoumarins in plants have been collected with methods that are nowadays considered outdated and very few papers based on a metabolomic approach are available. A more frequent recourse to novel extraction and analytical protocols should be encouraged, as well as a more intensive application of environmentally friendly strategies. Given the increasing relevance of synergistic effects in plant material used for health purposes and according to our increased capability of managing and decoding large datasets, metabolomic evaluations could be used more frequently also in combination with bioactivity assays. The recourse to metabolomics is to be considered relevant also from a toxicological standpoint, as intense CYP450 inhibitions have been reported, for instance, in food supplements also in the absence of most common furanocoumarins [153].

From a cultural point of view, instead, a lack of a multidisciplinary effort still hinders the research and it may affect in some occasions the quality of data collected. The limited consideration of the peculiar ecological role and of the biological accumulation of furanocoumarins in leaf surfaces is often overlooked in the development of extraction and analytical methods, thus leading to an underestimation of their presence in natural products, resulting in a difficult evaluation of their risk assessment. As dietary furanocoumarins are potentially harmful to consumer health, their precise and reliable quantification in food plants, food supplements, and pharmaceuticals should be considered a priority. 

Pharmacological and clinical studies focused on furanocoumarins have often been performed without pairing their results with a suitable quantification of putative active substances, thus producing results with limited repeatability and difficult interpretation by the scientific community. Finally, most of the literature regarding furanocoumarin bioactivity is focused on their effects against pathological conditions, with limited attention paid to their role as physiological modulators of health. The limited availability of investigations regarding healthy people represents a void in the field that, given the potency of these compounds and their presence in different plants, should also be taken into account. This may represent a valuable new field for furanocoumarin research, given also the request made by regulatory boards, like the European Food Safety Authority, in terms of evidence needed to obtain food claims.

All these aspects should be taken into careful consideration by both younger and trained researchers interested in the improvement of the science of plant-based natural products containing furocoumarins.

## Figures and Tables

**Table 1:** Compounds described in the present review article, and their biological activities discovered to date.

Compound	Biological Activity	References
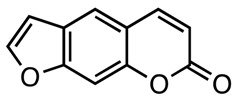	Photosensitizer, anti-proliferative and anti-hyperglycemic	[5,89,109,111,123]
Psoralen		
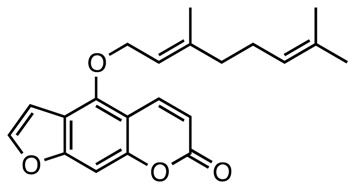	Cytoprotective, anti-proliferative, apoptotic agent, antibacterial, modulator of oral drug bioavailability, photosensitizer	[104,105,106,107,112,113,114,115]
Bergamottin		
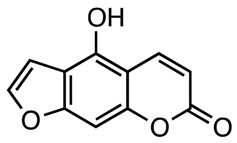	Antioxidant, anti-inflammatory, anti-proliferative, apoptotic agent	[112,120,133,134,137]
Bergaptol		
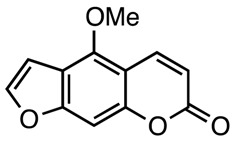	Photosensitizer, inducer of cell autophagy, antioxidant, anti-inflammatory, anti-proliferative, analgesic and anti-coagulant; bone health promotion enhancer	[89,112,117,118,119,120,125,132,136,137,138,139,143,144,145,146,147,148,149,150,151]
Bergapten		
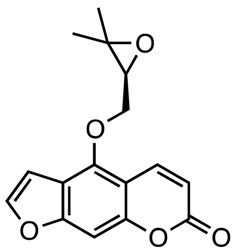	Antioxidant, anti-inflammatory, anti-proliferative, antibacterial, antifungal, antiarrhythmic, allelopatic and anti-estrogenic agent	[121,123,131,136,137,138,139]
Oxypeucedanin		
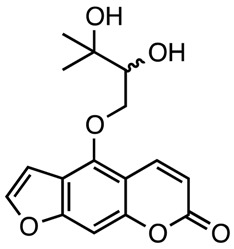	Antioxidant and anti-hyperglycemic	[123,131]
Oxypeucedanin hydrate		
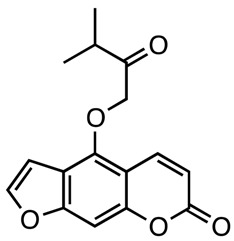	Anti-hyperglycemic	[123]
Isooxypeucedanin		
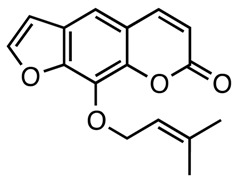	Antioxidant, GABA level enhancer, anticonvulsant drug action enhancer	[123,139,140,141,142]
Imperatorin		
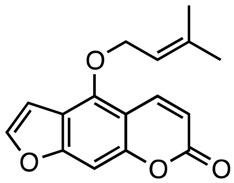	Antioxidant, anti-inflammatory, anti-proliferative, analgesic and anticoagulant	[131,136,137,138,139]
Isoimperatorin		
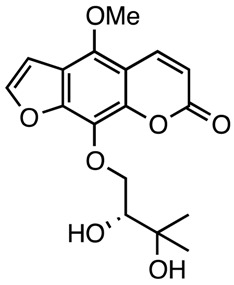	Antioxidant and anti-hyperglycemic	[123,131]
Byakangelicin		
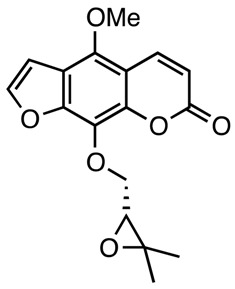	Antioxidant	[131]
Byakangelicol		
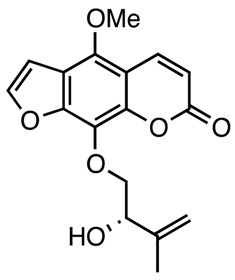	Antioxidant	[131]
Neobyakangelicol		
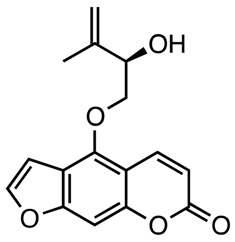	Anti-hyperglycemic	[124]
Pangelin		
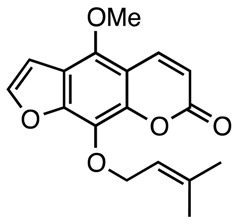	Antioxidant	[131]
Phellotorin		
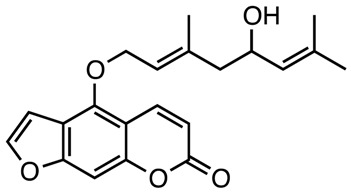	Anti-inflammatory, anti-proliferative, analgesic and anticoagulant	[136,137,138,139,140,141]
Notopterol		
